# Supplementary material for: Dose-Dependent Effects of Dietary Xylooligosaccharides Supplementation on Microbiota, Fermentation and Metabolism in Healthy Adult Cats
Source: Molecules. 2020 Oct 29;25(21):5030. doi: 10.3390/molecules25215030 (PMC7662210; doi:10.3390/molecules25215030)
Supplement: Supplementary file 1 [file molecules-25-05030-s001.pdf]

Supplementary Materials

Item	Min.	1st Qu.	Median	Mean	3rd Qu.	Max.	NA's
Faecal score	1.000	2.000	3.000	2.917	4.000	4.000	-
Gly	201.9	263.0	282.4	355.5	315.5	1486.2	1
Ala	346.2	492.9	554.9	595.1	710.7	900.1	1
Val	100.4	116.4	126.9	130.9	139.9	212.9	1
Leu	151.2	172.6	197.1	192.7	206.0	267.9	1
Orn	7.78	12.38	14.77	15.10	16.98	25.02	1
Met	28.23	33.21	40.45	40.64	47.09	64.80	1
Fen	58.84	73.61	82.82	80.34	86.86	101.55	1
Cit	8.87	20.34	24.68	25.53	30.14	47.71	1
Tyr	31.90	42.04	45.60	48.00	55.06	68.95	1
C0	9.57	15.84	19.76	22.91	25.38	67.36	1
C2	1.770	2.700	3.600	4.277	5.420	11.93	1
C3	0.040	0.130	0.180	0.184	0.210	0.430	1
C4	0.200	0.230	0.250	0.349	0.265	1.810	1
C5:1	0.010	0.030	0.030	0.037	0.040	0.110	1
C5	0.050	0.075	0.080	0.093	0.105	0.210	1
C6	0.000	0.080	0.095	0.113	0.130	0.320	-
3OH-C5	0.000	0.050	0.075	0.079	0.110	0.140	-
C8	0.000	0.030	0.040	0,052	0,050	0.370	-
C3DC	0.000	0.010	0.020	0.022	0.030	0.060	-
C10:1	0.000	0.010	0.015	0.018	0.020	0.090	-
C10	0.000	0.010	0.020	0.023	0.030	0.060	-
C4DC	0.000	0.038	0.050	0.048	0.060	0.100	-
C12	0.000	0.020	0.030	0.030	0.040	0.070	-
C6DC	0.000	0.000	0.010	0.008	0.010	0.020	-
3OH-C12	0.000	0.000	0.010	0.006	0.010	0.020	-
C14:2	0.000	0.010	0.020	0.017	0.020	0.040	-
C14:1	0.000	0.048	0.060	0.068	0.088	0.170	-
C14	0.000	0.030	0.040	0.042	0.050	0.090	-
C8DC	0.000	0.020	0.020	0.021	0.030	0.030	-
3OH-C14:1	0.000	0.010	0.015	0.017	0.020	0.050	-
3OH-C14	0.000	0.000	0.010	0.075	0.010	0.020	-
C16:1	0.000	0.040	0.050	0.053	0.070	0.120	-
C16	0.000	0.128	0.145	0.170	0.223	0.420	-
C10DC	0.000	0.010	0.010	0.014	0.020	0.030	-
3OH-C16:1	0.000	0.010	0.010	0.012	0.020	0.030	-
3OH-C16	0.000	0.000	0.010	0.006	0.010	0.020	-
C18:2	0.000	0.048	0.070	0.078	0.093	0.270	-
C18:1	0.000	0.148	0.160	0.193	0.233	0.580	-
C18	0.000	0.090	0.110	0.127	0.160	0.250	-
3OH-C18:1	0.000	0.008	0.010	0.010	0.010	0.020	-
3OH-C18	0.000	0.000	0.010	0.007	0.010	0.020	-
C24	0.000	0.010	0.020	0.020	0.030	0.040	-
C18DC	0.000	0.000	0.010	0.005	0.010	0.010	-

C26	0.000	0.000	0.010	0.008	0.010	0.020	-
Acetic acid	312.5	456.7	545.2	514.8	599.6	667.3	-
Propionic acid	118.6	176.3	241.8	227.2	263.2	380.6	-
Butyric acid	55.21	109.6	145.1	168.8	211.6	567.9	-
Valeric acid	12.32	55.80	85.42	81.30	98.75	201.5	-
Caproic acid	0.000	0.000	0.000	1.994	0.000	19.25	-
Isobutyrate	0.00	4.83	11.08	11.81	17.15	31.16	-
Isovalerate	0.00	16.23	23.68	26.86	36.98	62.39	-
Ammonia	0.100	0.160	0.205	0.213	0.260	0.370	-
Indol	1.42	7.17	17.97	29.58	43.77	158.9	2
Phenol	3.28	14.60	40.75	38.29	52.06	88.98	4
p_cresol	4.52	31.06	55.73	67.09	79.86	265.5	3

Table 1. Supplementary data.
